# Population shift in antibody immunity following the emergence of a SARS-CoV-2 variant of concern

**DOI:** 10.1038/s41598-025-89940-y

**Published:** 2025-02-14

**Authors:** Jinal N. Bhiman, Vimbai Sharon Madzorera, Qiniso Mkhize, Cathrine Scheepers, Tandile Hermanus, Frances Ayres, Zanele Makhado, Thandeka Moyo-Gwete, Carol Crowther, Beverley Singh, Mirriam Fortuin, Edmore Marinda, Sean Jooste, Khangelani Zuma, Nompumelelo Zungu, Lynn Morris, Adrian Puren, Leickness Simbayi, Sizulu Moyo, Penny L. Moore

**Affiliations:** 1 SAMRC Antibody Immunity Research Unit, School of Pathology, University of the Witwatersrand, Johannesburg, South Africa; 2 National Institute for Communicable Diseases of the National Health Laboratory Services, Johannesburg, South Africa; 3 Human Sciences Research Council, Pretoria, South Africa; 4 School of Public Health, University of the Witwatersrand, Johannesburg, South Africa; 5 School of Nursing and Public Health, College of Health Sciences, University of KwaZulu-Natal, Durban, South Africa; 6 Centre for the AIDS Programme of Research in South Africa, Durban, South Africa; 7 Department of Psychiatry and Mental Health, University of Cape Town, Cape Town, South Africa

**Keywords:** SARS-CoV-2, South Africa, Neutralizing antibodies, Antibody immunity, Population shift, Beta variant, Infectious diseases, SARS-CoV-2

## Abstract

Severe Acute Respiratory Syndrome Coronavirus 2 (SARS-CoV-2) variants of concern (VOCs) exhibit escape from pre-existing immunity and elicit variant-specific immune responses. In South Africa, the second wave of SARS-CoV-2 infections was driven by the Beta VOC, which coincided with the country-wide National COVID-19 Antibody Survey (NCAS). The NCAS was conducted between November 2020 and February 2021 to understand the burden of SARS-CoV-2 infection through seroprevalence. We evaluated 649 NCAS sera for spike binding and pseudovirus neutralizing antibodies. We classified individuals as ancestral or D614G neutralizers (114/649), Beta neutralizers (96/649), double neutralizers (375/649) or non-neutralizers (62/649). We observed a consistent decrease in preferential neutralization against the D614G variant from 68 to 18% of individuals over the four sampling months. Concurrently, samples with equivalent neutralization of both variants, or with enhanced neutralization of the Beta variant, increased from 32 to 82% of samples. Neutralization data showed that geometric mean titers (GMTs) against D614G dropped 2.4-fold, while GMTs against Beta increased 2-fold during this same period. A shift in population humoral immunity in favor of Beta-directed or cross-neutralizing antibody responses, paralleled the increase in genomic frequency of the Beta variant in South Africa. Understanding similar population immunity shifts could elucidate immunity gaps that drive SARS-CoV-2 evolution.

## Introduction

Following the global spread of Severe Acute Respiratory Syndrome Coronavirus 2 (SARS-CoV-2) in humans and the subsequent development of widespread population immunity, continual emergence of mutated variants with distinct antigenicity, transmissibility, and/or pathogenesis has been observed^1–11^. The emergence of SARS-CoV-2 variants of concern (VOCs) resulted in compromised sensitivity to neutralizing antibodies triggered in response to previous infection or vaccination^12–16^. South Africa was no exception to this and was the first country to report emergence of a constellation of mutations within the spike gene. With each epidemiological wave of SARS-CoV-2 infections in South Africa^17–19^, a different variant dominated^1–3^. The second wave started in October 2020 with a surge in cases in the Eastern Cape province, followed by a cascade of infections, first in other neighboring coastal provinces and then over the next two months, inland^1^. This second wave was caused by the B.1.351 lineage, which given its significant immune escape and increased transmissibility, was classified by the World Health Organization as the Beta VOC^1,12,15^.

While VOCs are themselves often resistant to immunity triggered by prior infection or vaccination, they also elicit neutralizing responses that vary in their titer and cross-reactivity to other variants^11,12,20–22^. For example, antibody responses elicited by D614G infection were unable to or showed reduced neutralization of the Beta variant due to the presence of the K417N, E484K and N501Y mutations in the spike’s receptor binding domain (RBD)^12^. However, we and others previously showed, in relatively small cohorts of hospitalized individuals, that Beta-elicited neutralizing antibodies had increased cross-reactivity for VOCs^15,21^. These studies were performed in real-time to inform public health policy, however whether these changes in neutralizing antibody specificity translated into detectable shifts in population immunity has only been inferred through genomic surveillance of symptomatic individuals, as well as assessment of neutralization sensitivity of emerging variants in small cohorts of previously infected and/or vaccinated individuals^12,16,21,23–25^.

In late 2020, the burden of asymptomatic SARS-CoV-2 was unknown globally and in South Africa, therefore the Human Sciences Research Council (HSRC) National COVID-19 Antibody Survey (NCAS) was initiated to measure SARS-CoV-2 seropositivity within households and estimate infection rates (regardless of symptoms) in South Africa following the first epidemiological wave of infections^26^. The first NCAS sampling period occurred across all provinces in South Africa from November 2020 to February 2021. This sampling period coincided with the first sequence detection of the Beta VOC in October 2020 and its relatively rapid expansion to dominance in South Africa by December 2020. Here, we assessed the binding and neutralizing capacity of sera collected during this transition phase between dominant SARS-CoV-2 variants to quantify changes in population immunity.

We show that as Beta came to dominate SARS-CoV-2 infections in South Africa, a concomitant shift to preferential neutralization of Beta occurred, with titers gradually increasing against Beta and waning against D614G. In addition, while cross-reactive D614G and Beta responses developed in the population, some individuals had more Beta-biased responses. Overall, this suggests that emerging VOCs, which replace previously circulating variants and become dominant, can alter immunity at the population level by eliciting cross-reactive response and/or variant-specific responses, the latter of which can create immunity gaps.

## Results

### Screening of individuals from the HSRC NCAS study for spike binding antibodies

**Fig. 1:**
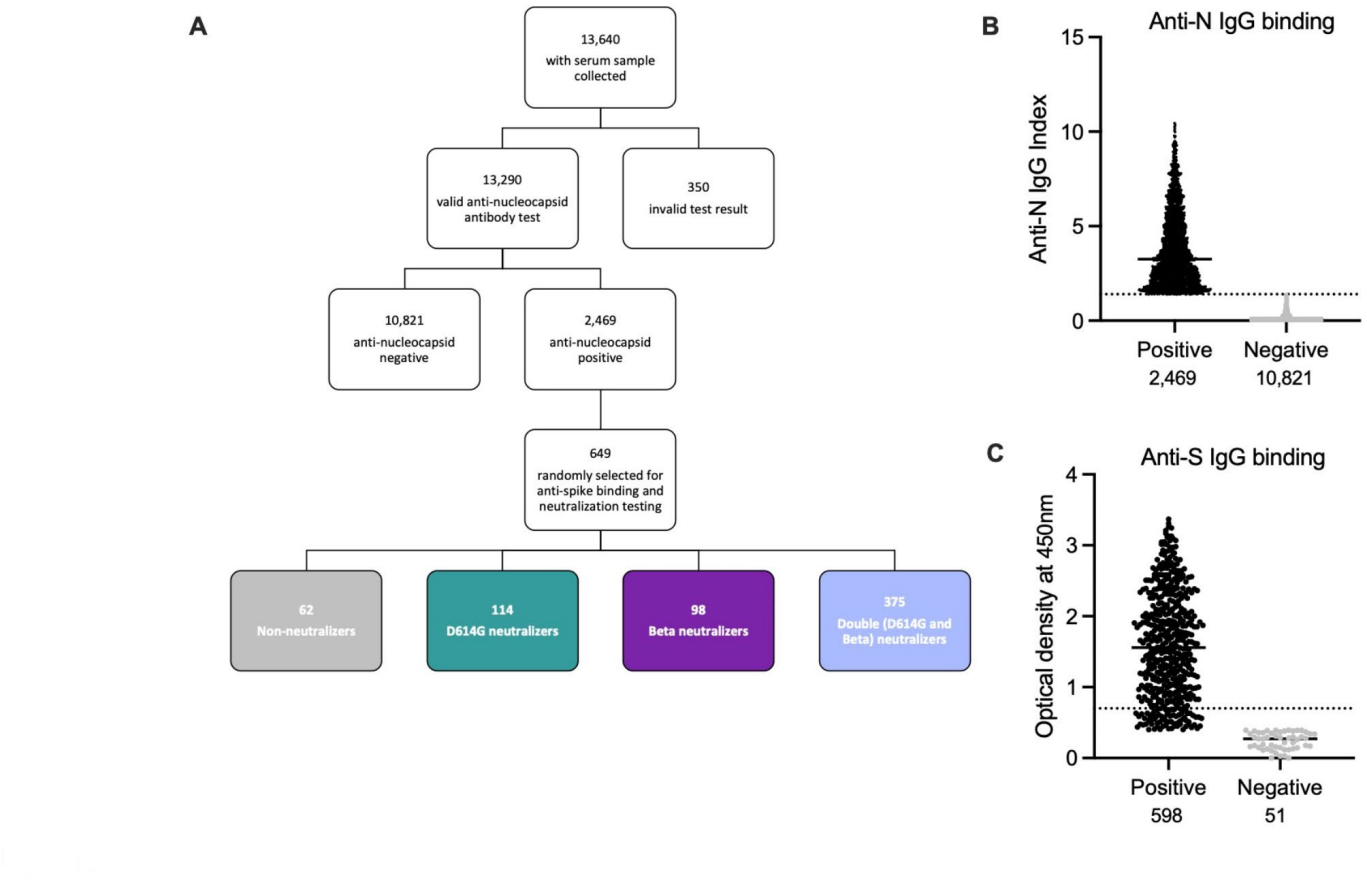
Screening of samples collected during the HSRC NCAS study for neutralization testing. (**A**) Flow diagram of samples prioritized according to anti-nucleocapsid and anti-spike antibodies for D614G and Beta neutralization testing. (**B**) Anti-nucleocapsid IgG binding by all tested plasma samples with the positivity cut-off IgG index value of 1.4 indicated by the dashed line. (**C**) Anti-spike IgG binding ELISA results for the samples that tested positive for anti-nucleocapsid IgG antibodies, with the anti-spike binding positivity cut-off of 0.4 IgG optical density (OD) at 450 nm indicated by a dashed line.

As part of the HSRC NCAS, serum samples from 13,640 individuals were collected, with 350 samples having an invalid anti-nucleocapsid binding antibody result (Fig. 1A). Of those with valid results, 18.5% (2,469/13,290) of the cohort was positive for anti-nucleocapsid antibodies indicating previous infection^26^ (Fig. 1A **and B**). This low anti-nucleocapsid seropositivity was consistent with (i) the strict lockdown measures implemented by the South African government to limit viral transmission and (ii) subsequent relatively low infection attack rates for both the D614G and Beta variants^17,19,26^. However, we cannot exclude the possibility of sero-reversion for the SARS-CoV-2 IgG chemiluminescent microparticle immunoassay (CMIA) used following a median of 242 days post-infection^27^. From these nucleocapsid positive individuals, a random selection, representing one third of the samples (649/2,469) was selected for detection of anti-spike trimer binding and neutralizing antibodies. Of these 649 individuals, 60.7% were female, 89.2% were black African, 20.5% were between 18 and 40 years of age and 70% of individuals were from urban areas (Table 1). Anti-spike trimer binding antibodies were detected in 92% (598/649) of the nucleocapsid-positive samples (Fig. 1C). These samples were tested for neutralization of the D614G and Beta variants, with 91% (587/649) of those sera having detectable neutralizing activity against at least one virus (Fig. 1A). Based on the neutralizing activity of these sera, we classified individuals as non-neutralizers (D614G and Beta ID_50_ < 20), D614G neutralizers (D614G ID_50_ > 20 and Beta ID_50_ < 20), Beta neutralizers ((D614G ID_50_ < 20 and Beta ID_50_ > 20) and double neutralizers (D614G and Beta ID_50_ > 20) (Fig. 1A; Table 2). These groups of non-neutralizers, D614G neutralizers, Beta neutralizers and double neutralizers made up 10% (62/649), 18% (114/649), 15% (96/649) and 57% (375/649) of all samples collected between November 2020 and February 2021 (Fig. 1A; Table 2).

**Table 1:** Demographic data of study population.

Variables	*n*	(%)
Gender		
Female	395	60.7
Male	255	39.2
Age		
12–17	95	14.6
18–30	157	24.2
31–40	106	16.3
41–50	97	14.9
51–60	82	12.6
61–70	74	11.4
71–80	30	4.6
>81	8	1.2
Race		
Black African	580	89.2
Coloured	59	9.1
White	5	0.8
Asian	5	0.8
Unknown	1	0.2
Locality type		
Urban area	461	71.0
Rural area	188	28.9

**Table 2:** Number of samples with equivalent or enhanced neutralization of the D614G and Beta variants.

Number of samples				
	Nov-20	Dec-20	Jan-21	Feb-21
Equivalent	35	96	110	44
Enhanced Beta	5	27	101	37
Enhanced D614G	85	69	66	18
Total samples	125	192	277	99

### Neutralization profiles as the Beta variant replaces D614G and begins to dominate

**Fig. 2:**
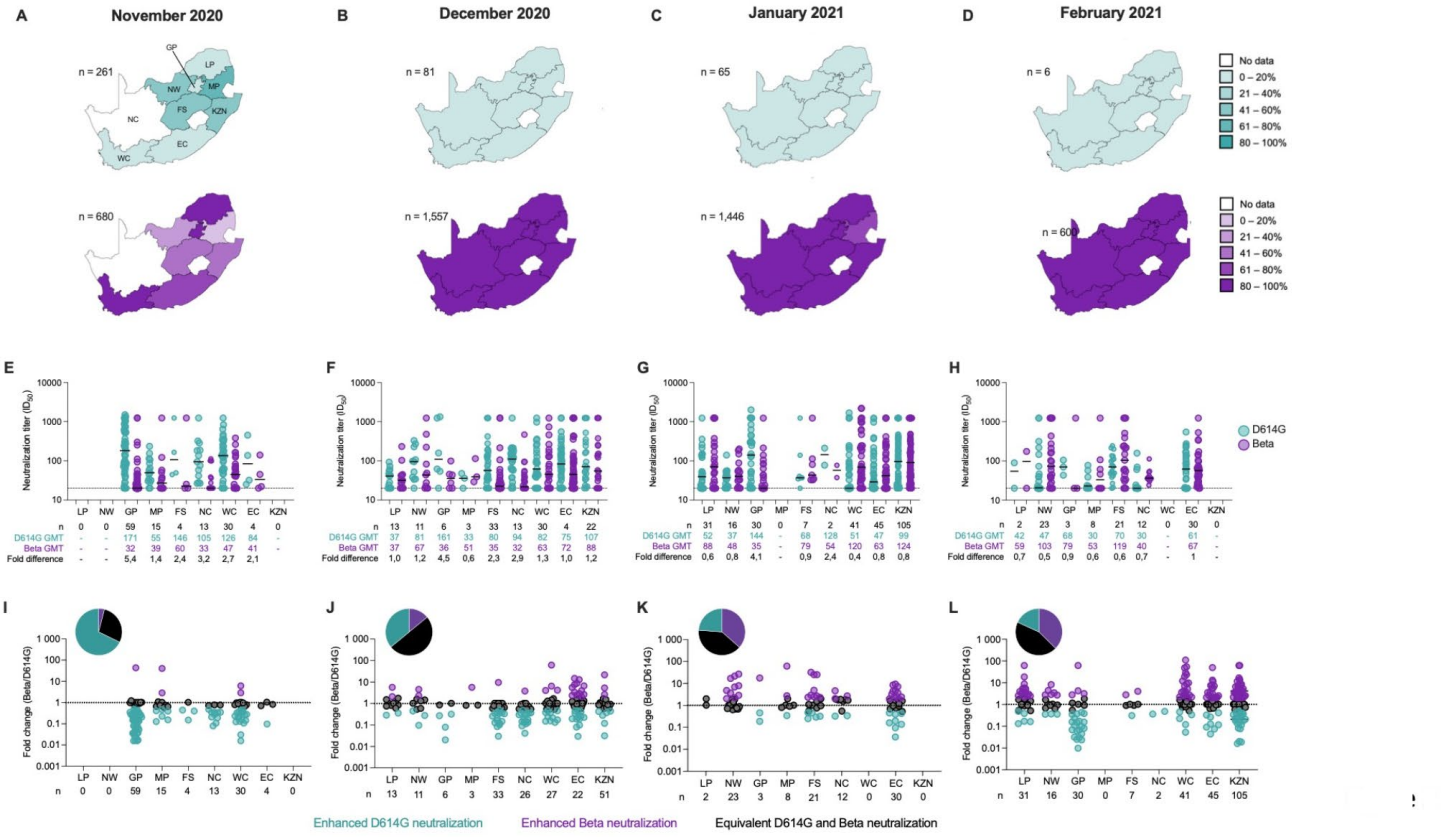
Neutralizing profiles of samples collected during and after the transition period from D614G to Beta dominance in South Africa. (**A**–**D**) Proportion of D614G (turquoise) and Beta (purple) genomes across the nine provinces of South Africa in November 2020 (**A**), December 2020 (**B**), January 2021 (**C**) and December 2021 (**D**). Frequency ranges of the respective genomes are indicated in different shades per the key and number of genomes for each month are indicated. (**E**–**H**) Neutralization titers against D614G (turquoise) and Beta (purple) across the nine provinces for November 2020 (**E**), December 2020 (**F**), January 2021 (**G**) and February 2021 (**H**). Geometric meant titers (GMT) for each virus as well as the number of samples tested in each province for each month are indicated. (**I**–**L**) Neutralization profiles for samples collected in November 2020 (**E**), December 2020 (**F**), January 2021 (**G**) and February 2021 (**H**) across the nine provinces. Samples with enhanced neutralization against D614G (turquoise), enhanced neutralization against Beta (purple) or equivalent neutralization against D614G and Beta (black) are indicated as a fold change in neutralization titer. The dashed line indicates no fold change in titer, with enhanced D614G and Beta neutralization located below and above the line respectively. Pie charts above each month of sampling summarizes all data for each respective month. Province names abbreviated as follows Gauteng (GP), Limpopo (LP), Mpumalanga (MP), North West (NW), Free State (FS), Northern Cape (NC), Western Cape (WC), Eastern Cape (EC) and KwaZulu-Natal (KZN).

We assessed South African national genomic surveillance data from the Global Initiative on Sharing All Influenza Data (GISAID) database^28^, for the frequency of SARS-CoV-2 ancestral-like D614G or Beta variants circulating between November 2020 to February 2021 (Fig. 2A-D; Tables 3 and 4). We compared genomic frequency of D614G and Beta to neutralization of both these variants by samples collected from each of the nine provinces (Fig. 2E-L; Table 5).

**Table 3:** Percent Beta genomes between November 2020 to February 2020 in South Africa.

Percent Beta Genomes				
Province	Nov-20	Dec-20	Jan-21	Feb-21
Eastern Cape	79	89	98	97
Western Cape	85	94	97	95
Gauteng	86	95	83	92
Free State	44	90	96	100
North West	29	81	91	100
Northern Cape	ND	89	93	96
Mpumalanga	20	90	75	100
Limpopo	100	94	92	100
KwaZulu-Natal	59	97	99	99

ND = no data.

**Table 4:** Percent D614G genomes between November 2020 to February 2020 in South Africa.

Percent D614G genomes				
Province	Nov-20	Dec-20	Jan-21	Feb-21
Eastern Cape	18	4	1	3
Western Cape	14	6	1	2
Gauteng	13	3	10	0
Free State	56	10	0	0
North West	56	11	0	0
Northern Cape	ND	11	1	2
Mpumalanga	70	7	0	0
Limpopo	0	0	0	0
KwaZulu-Natal	41	3	1	1

ND = no data.

**Table 5:** Geometric mean titers against D614G and Beta in single, double and non-neutralizers.

	GMT D614G	GMT Beta	*N*	Percentage
Nov-20				
Beta	NN	182	2	1,7
D614G	101	NN	51	42.1
Double	220	65	60	49.6
Non-neutralizer	NN	NN	8	6.6
Dec-20				
Beta	NN	63	14	4.5
D614G	56	NN	40	12.9
Double	141	89	232	75.1
Non-neutralizer	NN	NN	23	7.4
Jan-21				
Beta	NN	85	63	22
D614G	87	NN	44	15.8
Double	139	163	149	53.6
Non-neutralizer	NN	NN	22	7.9
Feb-21				
Beta	NN	64	27	27.3
D614G	44	NN	10	10.1
Double	90	124	56	56.6
Non-neutralizer	NN	NN	6	6.1

N = number of samples; GMT = geometric mean titer; NN = no neutralization.

In November 2020, D614G variants, including clades 19 A, 19B, 20 A, 20 C and 20D dominated in three provinces (Free State, North West and Mpumalanga), with the remaining provinces, for which data was available, showing 13–41% frequency of ancestral-like D614G variants (Fig. 2A; Table 3). In contrast, Beta variants dominated in five provinces (Eastern Cape, Western Cape, Gauteng, Limpopo and KwaZulu-Natal) with 20–44% in the Free State, North West and Mpumalanga provinces (Fig. 2A; Table 4). Geometric mean titers (GMTs) against D614G ranged between 84 and 171, however samples from Limpopo, North-West and KwaZulu-Natal provinces were not collected (Fig. 2E). Despite the significant differences in Beta dominance across the provinces, 1.4 to 5.4-fold lower GMTs against Beta (32 to 60) were observed in all provinces, including those where Beta accounted for more than 50% of genomes (Fig. 2E). We attributed this to a delay in the antibody response compared to detection of the variant at a population level. We therefore investigated differences in the quality of antibody response, by quantifying whether individuals showed enhanced or equivalent neutralization of D614G and Beta. The majority of serum collected from individuals in November 2020 displayed enhanced neutralization against the D614G variant (68%), with only 5 of 125 samples able to neutralize the Beta variant at a higher titer than the D614G variant and 28% of samples able to neutralize both viruses equivalently (Fig. 2E). These neutralization data clearly reflected the prior exposure of the population to the D614G variant, with relatively few infections caused by the Beta variant.

However, by December 2020, less than 11% of genomes in all provinces were ancestral-like (Fig. 2B), while the Beta variant dominated at 81–97%, with data available from all nine provinces (Fig. 2B). Overall samples collected during this month still showed higher GMTs against D614G (between 37 and 161) compared to Beta ( 32 to 88) in all provinces apart from Gauteng, although fold differences were smaller than in November 2020. During this month, an increase to 50% of samples that equivalently neutralized both the D614G and Beta variants was seen, while only 36% neutralized the D614G variant at higher titers (Fig. 2F).

Over the course of January and February 2021, the Beta variant continued to dominate, with some provinces displaying up to 100% of sequences belonging to this lineage (**Supp** Fig. 2C-D). During these months, we saw a significant shift in immunity, with GMTs against Beta higher than D614G in most provinces (5/7 for January and 6/7 for February) (Fig. 2G-H). The frequency of samples displaying higher neutralization of the D614G variant further decreased to 24% (*n* = 66/277) and then 18% (*n* = 18/99) of samples, while those with higher titers against the Beta variant increased to 36% (*n* = 100/277) and 37% (*n* = 37/99) in January and February respectively (Fig. 2K-L**)**. In addition, samples that were able to neutralize both variants equivalently remained at a somewhat stable level between 40 and 44% for these two months (Fig. 2K-L).

### Population immunity shift is observed as Beta outcompetes D614G variants

**Fig. 3:**
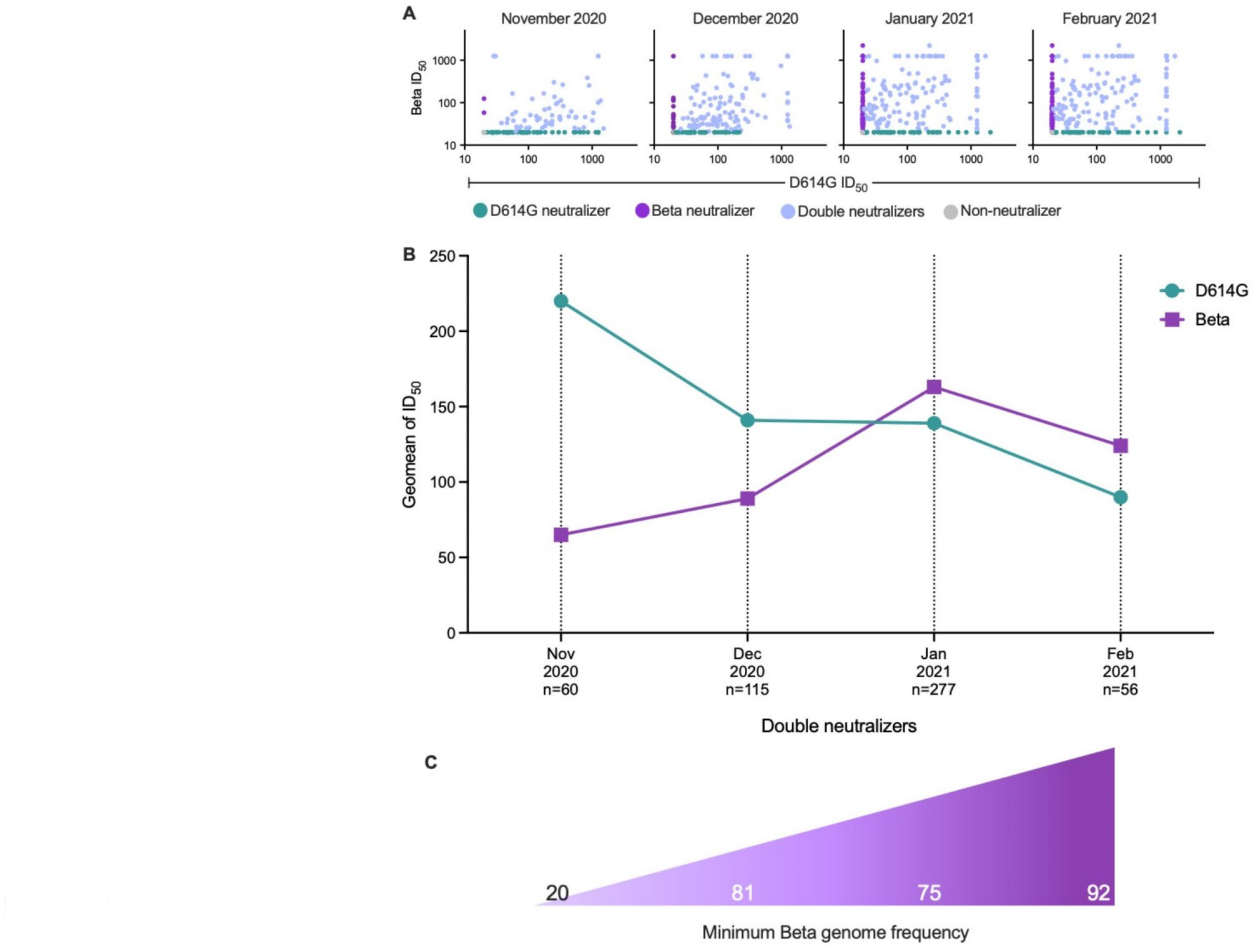
Concurrent population shift in neutralizing immunity with the expansion to dominance of the Beta variant. (**A**) Neutralization titers (represented as inhibitory dose at 50% reduction of signal, ID_50_) of the D614G and Beta variants. Samples classified as D614G (turquoise), Beta (purple), double neutralizer (blue) or non-neutralizer (grey) over November 2020, December 2020, January 2021 and February 2021. (**B**) Geometric mean titers (GMT) of the double neutralizers (with the capacity to neutralize both the D614G and Beta variants were) are displayed over the sampling period. F-statistic and p value are displayed and were calculated using F-test for linear regression slopes. (**C**) Number of samples tested and the minimum frequency of Beta sequences for each month displayed.

As previously described, we had categorized samples collected from November 2020 to February 2021, according to their neutralization profiles as Beta, D614G, double or non-neutralizers (Fig. 1A). We compared the D614G and Beta variant neutralization titers within each group, regardless of the province, over the same sampling period (Fig. 3A-C). We observed a noticeable shift in immune profiles with a consistent increase in the frequency of double (49,6% to 56,6) and Beta neutralizers, coupled with a concomitant decrease in the frequency of D614G neutralizers (42,1% to 10,1%) (Fig. 3A-C).

Additionally, as a more quantitative measure, we evaluated changes in the geometric mean titers (GMT) against the D614G and Beta variants over time in the double neutralizing group (Fig. 3B). In alignment with our prior analyses, we saw 3.4-fold higher titers against D614G compared to Beta during November 2020, with these titers dropping consistently over the next three months to a GMT of 99 against D614G. Conversely the GMTs against the Beta variant increased 2-fold from 65 to 124 between November 2020 and February 2021 (Fig. 3B**)**. To quantify the relationship between either D614G or Beta antibody titer and genome frequency, we used linear regression and defined the difference between these slopes using an F-test, which demonstrated a strong statistically significant difference in the slopes between D614G and Beta (F = 55, Dfn = 1, DFd = 4 and *p* = 0,0017). Titers against the Beta variant were modest, with a GMT of 66, 86, 135, and 100 in each of the four sampling months (November 2020 to February 2021), consistent with the high rate of asymptomatic infections in South Africa^29^.

## Discussion

We assessed the changes in the neutralizing capacity of sera from individuals in multiple provinces of South Africa over a sampling period when the Beta variant came to dominate and replaced the ancestral/D614G variants. Previous studies using relatively small infection cohorts^12,15,20^ or in vaccine trial participants^13,30,31^, showed that despite there being only 7–10 spike substitutions in Beta compared to ancestral variants, neutralizing antibody responses were profoundly different, with little to no cross-reactivity to Beta in individuals exposed to the ancestral spike. Our study confirmed these findings, and extended this to a general population setting, showing that individuals with D614G exposures were more likely to exhibit neutralizing activity against this variant only. In addition, we previously showed that in hospitalized individuals, more cross-reactive responses capable of targeting the D614G and Beta variant were elicited by Beta infections compared to D614G infections^21^. Here we again provide support for this, but in the context of mild/asymptomatic infection where, as Beta infections increased to dominance, so too did the fraction of samples with cross-reactivity for D614G and Beta. In a household transmission study investigating the contribution of neutralizing antibodies as correlates of protection, a relatively low GMT of 146 was shown to mediate 37% protection from reinfection by the Delta variant^32^. This suggests that the relatively low D614G and Beta titers we report here may provide significant selection pressure on subsequent variants. However, given that we do not have infection histories, we cannot exclude the possibility that the increase in the proportion of cross-reactive responses in this cohort may have resulted from secondary Beta infections following primary D614G infection. Waning of antibody responses (which we cannot account for without infection history) may also have impacted on GMTs in the later sampling months of January and February 2021, given that the half-life of primary infection-induced immunity has been shown to be 121 days^32^. In addition, sampling of individuals infected with Beta was likely closer to the time since infection as compared to those infected with D614G, which may confound the interpretation of GMT and cross-reactivity. Another limitation of our study is that, despite the breadth of coverage of samples collected from multiple provinces over this period, we did not have balanced sampling across the provinces, nor sampling that reflected provincial population sizes or rates of SARS-CoV-2 infection per province. We therefore did not see statistically significant differences in neutralization profiles between provinces at each time point, making it impossible to correlate the geographic spread of the virus with the subsequent immunity shift at provincial resolution. For example, in the Eastern Cape, where the Beta variant first emerged, relatively few samples were collected in November 2020 from this province. This study provides serological support for previous findings from genomic surveillance of SARS-CoV-2, in that exposure to different SARS-CoV-2 variants, here in an unvaccinated population, can contribute to increasing cross-reactivity of immune responses to multiple variants, but can also result in variant-specific responses, thereby generating immunity gaps, which can be exploited in SARS-CoV-2 evolutionary patterns.

## Methods

### Cohort

The HSRC NCAS study was carried out from December 2020 to February 2021, over two rounds of data collection in all nine provinces in South Africa^26^. The survey targeted individuals older than 12 years of age, in households within selected geographic small area layers (SALs) stratified by province.

### Ethics approval and consent to participate for use of human participants

The Human Sciences Research Council (HSRC) Research Ethics Committee approved the survey protocol (REC 4/17/06/20) and all experiments were performed in accordance with the HSRC Research Ethics Committee guidelines and regulations, which are in accordance with the Declaration of Helsinki. Verbal or written informed consent was required for survey participation. Informed consent was obtained from legal parents or guardians for all Minor participants. In addition to the data collected using household and individual questionnaires, venous blood samples were collected from 13,640 consenting participants.

### SARS-CoV-2 anti-nucleocapsid binding antibody immunoassay

Antibodies to SARS-CoV-2 nucleocapsid in plasma samples was measured using the SARS-CoV-2 IgG chemiluminescent microparticle immunoassay (CMIA), Abbott Ireland, Diagnostics Division, Finisklin Business Park, Sligo, Ireland, and run on the Abbott Architect I1000SR instrument as previously described^26^. Briefly, the sample was incubated with SARS-CoV-2 nucleocapsid coated paramagnetic microparticles for 18 min. These beads were then subjected to a series of washes following which an anti-human IgG acridinium-labelled conjugate was added. Following incubation and another wash cycle, the pre-trigger was added resulting in chemiluminescent quantified as relative light units (RLU) and expressed as calculated index (RLU of sample normalized to RLU of the calibrator). A sample to calibrator (S/C) index of greater than 1.4 was interpreted as a positive result. Assay sensitivity as demonstrated by the manufacturer post onset of symptoms is 25% at 3–7 days, 86.36% at 8–13 days and 100% at ≥ 14 days, but with a median sero-reversion rate of 242.5 days^27^. Specificity is 99.63% as per manufacturer.

### SARS-CoV-2 anti-spike antibody assay

An enzyme-linked immunosorbent assay (ELISA) was performed to assess the presence of SARS-CoV-2 spike-specific antibodies binding in participant samples as previously described^12^. Recombinant SARS-CoV-2 spike D614G HexaPro^33^ protein was coated at 2 µg/ml onto 96-well, high-binding plates and incubated overnight at 4 °C. The plates were then washed and incubated at 37 °C for 2 h in blocking buffer (5% skimmed milk powder, 0.05% Tween 20, 1× phosphate buffered saline). Plasma samples were added at 1:100 dilution and serially diluted three-fold in blocking buffer. Following a 1 h 30 min incubation at 37 °C, an anti-human horseradish peroxidase-conjugated antibody was added for 1 h at 37 °C. The signal was developed with TMB substrate (Thermo Fisher Scientific) for 5 min at room temperature, followed by addition of 1 M H_2_SO_4_ stop solution. Absorbance at 450 nm was measured and reported as an optical density.

### Lentiviral pseudovirus neutralization assay

SARS-CoV-2 specific neutralizing antibodies were measured using lentiviral pseudoviruses, as described^12^. Confluent cell monolayers of HEK 293T cells engineered to over-express human angiotensin converting enzyme 2 (hACE2) receptors (kindly provided by Michael Farzan (Scripps Research), were prepared in Dulbecco’s Modified Eagle Medium (DMEM, Gibco BRL Life Technologies) enriched with 10% heat inactivated fetal bovine plasma (FBS), 2.5% HEPES, 5% gentamicin, and 3 µg/ml puromycin at 37 °C 5% CO_2_. A solution of 0.25% trypsin in 1mM EDTA (Gibco BRL Life Technologies) was used to discrupt confluency of the cellular monolayer. The SARS-CoV-2 Wuhan-1 spike was cloned into the pCDNA 3.1 plasmid and then mutated using the QuickChange Lightning Site-Directed Mutagenesis kit (Agilent Technologies) and NEBuilder HiFi DNA Asembly Master Mix (NEB) to introduce the D614G mutation or mutations defining the Beta lineage (substitutions in the receptor binding domain (RBD - K417N, E484K, and N501Y) and changes in the N-terminal domain (NTD - L18F, D80A, D215G, and R246I). For the production of pseudoviruses, the 293T/17 cells were co-transfected with a lentiviral backbone (HIV-1 pNL4.Luc encoding the firefly luciferase gene) and either of the SARS-CoV-2 spike plasmids with PEIMAX (Polyscience). Culture supernatents were clarified of cells by passing through a 0.45 μm filter and stored at -70 ºC. Plasma samples were heat-inactivated at 56 ºC and clarified by centrifugation at 14,000 rpm for 2 min. Serially diluted plasma samples were incubated with pseudovirus for 1 h at 37 ºC, 5% CO_2_. 1 × 10^4^ cells per well were added after the 1 h incubation and incubated for a further 72 h. After the 72 h incubation, luminescence was measured using the PerkinElmer Life Sciences Model Victor X luminometer. Neutralization was measured as described by a reduction in luciferase gene expression after a single-round infection of 293T/ACE-2.MF cells with spike pseudotyped lentiviruses. Viral titers were calculated as the reciprocal plasma dilution (ID_50_) causing 50% reduction of relative light units.

### Analysis

We performed statistical analyses and graphical presentations using GraphPad Prism v9.1.0 (GraphPad Software, LLC) and the R statistical programming language (version 4.1.0) with the following packages: maptools, ggplot2, tidyverse, sf, rnaturalearth, rnautralearthdata and rgdal.

## Data Availability

All data generated or analyzed during this study are available upon request. The findings of this study are based on Nextstrain clade assignments associated with 4,839 sequences available on GISAID up to August 16, 2022, and accessible at 10.55876/gis8.240916uz.
